# Simultaneous bilateral angioplasty and stenting for carotid stenosis – a single center experience

**DOI:** 10.25122/jml-2021-0274

**Published:** 2022-02

**Authors:** Dmytro Viktorovych Shchehlov, Oleg Yevgenovych Svyrydiuk, Mykola Bohdanovych Vyval, Olena Fedorivna Sydorenko, Nataliia Mykolayivna Nosenko, Maxym Stepanovych Gudym

**Affiliations:** 1. State Organization Scientific-Practical Center of Endovascular Neuroradiology, National Academy of Medical Sciences of Ukraine (NAMS), Kyiv, Ukraine

**Keywords:** bilateral carotid artery stenosis, simultaneous bilateral angioplasty, stenting

## Abstract

Carotid artery stenosis is responsible for up to 12% of all ischemic strokes. The prevalence of bilateral carotid artery stenosis is nearly 8–39% among patients with stroke, and its management is still controversial. This study aimed to report the treatment results of bilateral carotid artery stenosis with simultaneous bilateral angioplasty and stenting (sbCAS) in a single institution during the last 10 years. 315 patients underwent carotid stenting in the Scientific-Practical Center of Endovascular Neuroradiology, NAMS of Ukraine during 2010–2020. 39 (12.4%) patients (mean age 57.9±2.1 – 28 men) underwent sbCAS. Primary clinical endpoints (stroke, myocardial infarction, or death) and secondary endpoints (hemodynamic depression (HD) – hypotension (<90 mmHg) or bradycardia (<60 bpm) and hyperperfusion syndrome (HPS) were evaluated. All sbCAS were technically successful, and a reduction of stenosis was noted in each case. There were two periprocedural neurological complications, one transient ischemic attack (TIA), and one minor stroke with the Modified Rankin Scale (mRS) – 3 at discharge. No myocardial infarction (MI) or death during hospitalization was noted. 28 patients (71.8%) had HD, and 2 (5.1%) had HPS. All patients except those with periprocedural stroke were discharged or transferred to another hospital without neurological deterioration. sbCAS is an effective and relatively safe procedure for carefully selected patients with bilateral carotid stenosis. Patients with bilateral carotid stenosis should be carefully examined, and the best treatment strategy should be assessed using a multidisciplinary approach taking into account the possibility of sbCAS.

## Introduction

Stroke is a leading cause of death worldwide, with a prevalence of nearly 13.7 million people every year. Approximately 80% of strokes are ischemic, and although the prevalence is growing among cerebrovascular diseases, its mortality and morbidity decreased during the last decade [1]. Carotid artery stenosis is responsible for up to 12% of all ischemic strokes. Recent data support surgery carotid endarterectomy (CEA) or carotid angioplasty and stenting (CAS) for patients with symptomatic carotid stenosis. However, the best management of carotid stenosis is still under investigation, although being meticulously studied in a few large, randomized control trials [2]. CEA and CAS have their advantages and disadvantages, and only careful selection of the patients with important preoperative planning can tailor the choice. However, challenges still exist in patient selection and the best timing for revascularization. Treatment of bilateral carotid disease with nearly 8–39% prevalence among patients with symptomatic carotid stenosis is one of the most undiscussed questions. Bilateral carotid stenosis is still a relative contraindication to CEA and is excluded from most prospective trials [3].

Nevertheless, patients with bilateral carotid stenosis and a high risk of stroke can succeed with well-timed revascularization. With recent advances in endovascular technology, simultaneous bilateral angioplasty and stenting (sbCAS) was described as a safe and effective treatment strategy, with risks of complications compatible with unilateral CAS. This article aims to report the results of sbCAS for bilateral carotid stenosis.

## Material and Methods

315 patients who underwent carotid stenting in the Scientific-Practical Center of Endovascular Neuroradiology, NAMS of Ukraine during 2010–2020 were evaluated. Among them, 39 (12.4%) patients (mean age 57.9±2.1; 28 – men) underwent sbCAS; the other 223 patients received unilateral CAS and were excluded.

37 patients among the sbCAS group were symptomatic at least on one side, and 2 patients underwent CAS before elective cardiac revascularization. The clinical characteristics of patients included were retrospectively analyzed according to collected data and are finalized in Table 1.

**Table 1.** Characteristics of the patients who underwent simultaneous bilateral carotid angioplasty and stenting.

**Patient characteristics **	**N of patients (%)**
**Male/female**	28/11
**Age, y**	57.9±2.1
**Clinical presentation TIA Stroke Asymptomatic**	1 13 (33.3%) 24 (61.5%) 2 (5.2)
**Hypertension**	24 (61.5%)
**Diabetes mellitus**	15 (38.5%)
**Smoking**	34 (87.2%)
**Myocardial infarction**	4 (10.3%)
**Heart failure **	8 (20.5%)

### Pre – CAS evaluation and patient selection

**Figure 1.**
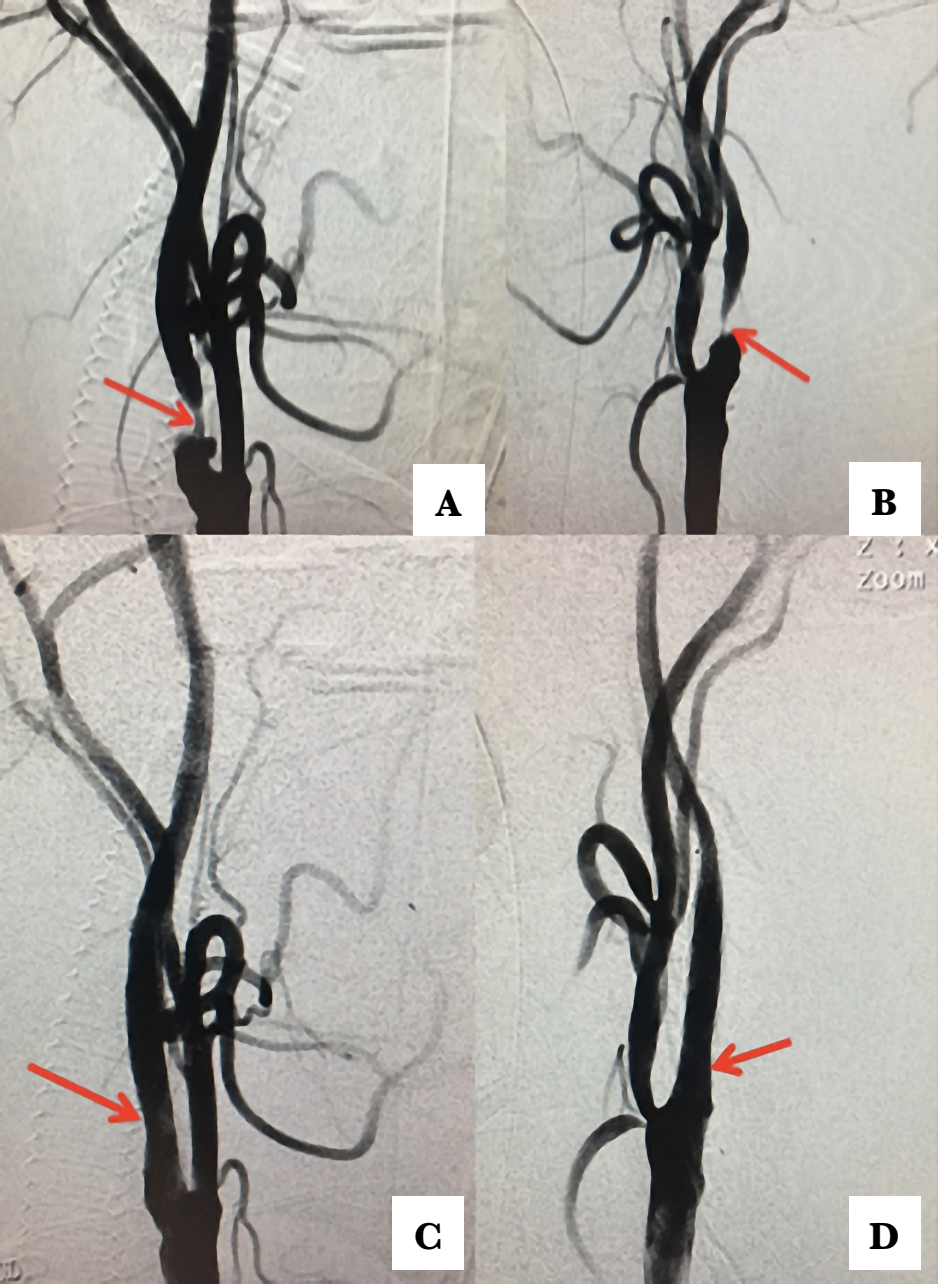
DSA before (A and B) and after (C and D) sbCAS in a patient with severe critical symptomatic carotid stenosis. The postoperative period was unremarkable, and the patient was discharged on day 3 after surgery.

All patients underwent digital subtraction angiography before stenting. The indication for CAS was based on the North American Symptomatic Carotid Endarterectomy Trial measurement [4]. The side of carotid stenosis was considered symptomatic if there was a transient ischemic attack or a non-disabling stroke before admission, according to CT and/or MRI findings. The indication for revascularization was carotid diameter reduction >50% on a symptomatic side and >70% on the asymptomatic side (Figure 1 A and B). Two asymptomatic carotid plaques with less than 70% were considered as high risk according to carotid ultrasound data. After signing the consent statement, all patients received antiplatelet medication (clopidogrel 75 mg per day and aspirin 100 mg per day for at least 5 days before the procedure). All lipid-lowering and anti-hypertensive agents continued before and after CAS, except beta-blockers in the morning of the procedure.

Since 2018 before CAS, clopidogrel sensitivity using light transmission aggregometry with ADP 5 μmol/L was measured in all cases. Clopidogrel resistance was determined with a maximum aggregation >50% on the aggregation curve. In the case of clopidogrel resistance, the daily and loading doses were increased to 150 and 450 mg, respectively.

### CAS Procedure

All CAS procedures were performed via femoral access under conscious sedation or general anesthesia. An 8F introducer sheath was placed in the femoral artery, and 5000 U of heparin was administered. Under fluoroscopy, an 8F guiding catheter [Envoy (Cordis Neurovascular, Miami Lakes, Fla)] was navigated proximal to the site of stenosis on the symptomatic side, and then a distal embolic protection device was placed distally [Filterwire EZ (Boston Scientific, MA, USA) or Spider RX (Medtronic/Covidien, Minneapolis, USA)] EmPro (MicroVention, Tustin, California, USA). In case of severe stenosis ≥80%, pre-dilatation with balloon (3–5 mm) Lovex (Balton Products, Warsaw), Submarine (Medtronic, Minneapolis, MN, USA) was performed before stent implantation. After the pre dilatation self-expanding stents, Precise RX (Cordis Corporation) and Protégé RX (Medtronic/Covidien, Minneapolis, USA), MER (Balton, Poland), and WallStent (MicroVention, Tustin, California,USA) were placed in the site of stenosis. After a control angiographic run, if the residual stenosis ≥30% was noted, post dilatation with a balloon (5–7 mm) Lovex (Balton Products, Warsaw), Submarine (Medtronic, Minneapolis, USA) was performed. After a successful procedure, the distal embolic protection device was repositioned into another ICA distally to the stenotic side, and the same procedure was repeated (Figure 1).

Heart rate and blood pressure were carefully monitored during the procedure, and Atropine (0.5–1.0 mg) was given to avoid or attenuate bradycardia. Severe hypotension was treated with IV infusions and small doses of vasopressors (dopamine or noradrenaline). All patients after the simultaneous procedure were neurologically and hemodynamically monitored in the ICU for at least 12 hours to check their neurological state, maintain a normal level of heart rate and blood pressure. After discharge, all patients continued double antiplatelet therapy, lipid-lowering, and anti-hypertensive agent.

### Clinical Endpoints

Any stroke, myocardial infarction, or death were considered the primary endpoint. Their appearance was noted during the procedure and follow-up period. The secondary endpoints included HD (hypotension (<90 mm Hg) or bradycardia (<60 bpm) and hyperperfusion syndrome (HPS) (severe headache with or without nausea or vomiting, focal seizures, or neurological deficit with CT/MRI negative findings). The incidence of the primary and secondary endpoints within the follow-up period was considered in the outcome measurement. All values were expressed as mean SD.

## Results

All sbCAS were technically successful, and a reduction of stenosis was noted in each case (Figure 1 C and D). Procedural details are shown in Table 2.

**Table 2.** Procedural data of simultaneous bilateral carotid angioplasty and stenting.

**Characteristics**	
**Simultaneous bilateral CAS (%)**	39 patients/78 arteries (100%)
**Length of stenosis (mm)**	15.3±2.9
**Stenosis location Common-ICA ICA**	20 (25.6%) 58 (74.4%)
**Degree of stenosis <50% 50–69% >70%**	0 36 (46.2%) 42 (53.8%)
**Ulceration**	21 (26.9%)
**Calcification**	27 (34.6%)
**Predilatation**	41 (52.6%)
**Neuroprotection device**	78 (100%)
**Postdilatation**	78 (100%)
**Total stented length, mm**	37.8±5.7
**Time of procedure (min)**	62±18
**Fluoroscopy time (min)**	19.5±4.2
**Mean contrast dose (ml)**	129±15.2

### Primary and secondary endpoints

We observed two periprocedural neurological complications, one TIA and one minor stroke due to the distal M-4 occlusion despite neuroprotection device, with mRS-3 at discharge and no major stroke. No MI or deaths were seen among patients who underwent sbCAS during hospitalization.

28 patients (71.8%) had hemodynamic depression after balloon dilatation. Two of them required vasopressors and prolonged ICU staying. Two patients (5.1%) had HPS, and one had neurological decline just after the sbCAS. The CT scan was unremarkable. Furthermore, another patient had agitation with personal changes. Both were carefully monitored in the ICU and recovered well after HPS. There was no case of mortality during the hospital stay. All complicated cases were carefully analyzed and summarized in Table 3.

**Table 3.** Complications of simultaneous bilateral carotid angioplasty and stenting.

**Characteristic**		**TIA/Stroke (2 pts)**	**Hd (28 pts)**	**HPS (2 pts)**
**Male/female**	28/11	2/0	19/9	2/0
**Age, y**	57.9±2.1	62.5±3.9	67.9±2.1	56.4±3.2
**Clinical presentation** **TIA** **Stroke**	15 24	0 2 (100%)	9 (32.1%) 19 (67.9%)	0 2 (100%)
**Hypertension**	24	2 (100%)	13 (46.4%)	1 (50.0%)
**Diabetes mellitus**	15	0	6 (21.4%)	1 (50.0%)
**Smoking**	34	2 (100%)	23 (82.1%)	2 (100%)
**History of myocardial infarction**	4	0	2 (7.1%)	0
**Heart failure **	8	0	2 (7.1%)	0
**Length of stenosis (mm)**	15.3±2.9	14.1±2.4	16.1±3.4	13.4±1.7
**Degree of stenosis** **<50%** **50–69%** **>70%**	0 36 (46.2%) 42 (53.8%)	0 1 (50.0%) 1 (50.0%)	0 8 (28.6%) 20 (71.4%)	0 0 2 (100%)
**Ulceration**	21	2 (100%)	4 (14.3%)	1 (50.0%)
**Calcification**	27	0	22 (78.6%)	0
**Total stented length, mm**	37.8±5.7	40.1±3.7	38.2±3.2	37.5±4.2
**Time of procedure (min)**	62±18	74±17	59±14	61±17
**Fluoroscopy time (min)**	19.5±4.2	31.3±6.1	17.3±3.1	18.6±6.2
**Mean contrast dose (ml)**	129±15.2	160±25.3	112±16.3	115±13.3

## Discussion

Bilateral carotid stenosis is a challenging pathology, and the optimal treatment strategy is still debated nowadays as there are few feasible approaches to treat these patients: staged or simultaneous CEA or CAS or its combination [5–12]. However, the best one has to be defined on an individual basis, considering symptoms, type of plaque, aortic arch, ICA anatomy, and comorbidities [5].

Fast improvement of endovascular approach and introduction of the distal embolic prevention devices and stents design made CAS a reasonable option for patients with bilateral stenosis, with a lower incidence of cranial nerve palsy and MI, considering that simultaneous bilateral CEA carries a risk of severe complications due to phrenic, pharyngeal and vagus nerve injury [6]. Most vascular surgeons would prefer staged CEA for the second stage due to concern about hemodynamic impairment from stimulation of the carotid sinus after stenosis correction and the risk of cerebral hyperperfusion syndrome [13]. However, a 2-staged procedure also increases nerve injury and non-neurological complications after two CEA [14].

During the last decade, more and more data support sbCAS in treating bilateral carotid stenosis as a safe and effective procedure [7, 8, 11, 12, 14–16]. Few concerns argue simultaneous procedure: high-risk surgical candidate for CEA; cases with severe concurrent diseases that require surgeries; severe contralateral carotid stenosis can cause new stroke after persistent HD and hypotension after the procedure; expenses of two procedures, that are sometimes critical, especially in low and middle-income countries [17]. On the other hand, the risk of bradycardia, hypotension, and HPS is higher during sbCAS [10].

A meta-analysis of sbCAS by Lai Z. *et al.* revealed that only hyperperfusion syndrome, 3.33% (95% CI, 1.66–5.55%), was higher after the combined procedure compared with unilateral CAS. Hemodynamic depression, 46.12% (95% CI, 33.16–59.35%), stroke, 3.20% (95% CI, 1.59–5.36%), myocardial infarction (MI), 0.60% (95% CI, 0.00–1.43%), and death, 1.20% (95% CI, 0.03–2.38%) were comparable to unilateral CAS [16]. There were no cases of MI or death in our series; however, periprocedural complications were seen, and in one case, it was disabling.

Our present study has some limitations. First, it is a retrospective study without a control group. Our data support the strategy of sbCAS in carefully selected patients; however, further well-designed prospective studies are necessary to evaluate the safety and cost-effectiveness compared with the staged procedure.

## Conclusions

SbCAS is a relatively safe and effective procedure for carefully selected patients with bilateral carotid stenosis and can decrease the risk of repeated cerebrovascular events. Therefore, patients with bilateral carotid stenosis should be carefully examined, and the best treatment strategy should be evaluated using a multidisciplinary approach considering the possibility of sbCAS.

## Acknowledgments

### Conflict of interest

The authors declare no conflict of interest.

### Authorship

DS, OS, NN contributed to conceptualization and methodology. OS, MV, MG, OS contributed to the investigation, data curation, formal analysis, and visualization. DS, OS, NN contributed to supervision and validation. All authors contributed to writing the original draft, reviewing, and editing the manuscript.
